# De novo resveratrol production through modular engineering of an *Escherichia coli*–*Saccharomyces cerevisiae* co-culture

**DOI:** 10.1186/s12934-020-01401-5

**Published:** 2020-07-14

**Authors:** Shuo-Fu Yuan, Xiunan Yi, Trevor G. Johnston, Hal S. Alper

**Affiliations:** 1 Institute for Cellular and Molecular Biology, The University of Texas at Austin, Austin, TX USA; 2 Department of Chemistry, University of Washington, Box 351700, Seattle, WA USA; 3 McKetta Department of Chemical Engineering, The University of Texas at Austin, 200 E Dean Keeton St. Stop C0400, Austin, TX 78712 USA

**Keywords:** Resveratrol, Modular metabolic engineering, Synthetic co-culture system

## Abstract

**Background:**

Resveratrol is a plant secondary metabolite with diverse, potential health-promoting benefits. Due to its nutraceutical merit, bioproduction of resveratrol via microbial engineering has gained increasing attention and provides an alternative to unsustainable chemical synthesis and straight extraction from plants. However, many studies on microbial resveratrol production were implemented with the addition of water-insoluble phenylalanine or tyrosine-based precursors to the medium, limiting in the sustainable development of bioproduction.

**Results:**

Here we present a novel coculture platform where two distinct metabolic background species were modularly engineered for the combined total and de novo biosynthesis of resveratrol. In this scenario, the upstream *Escherichia coli* module is capable of excreting *p*-coumaric acid into the surrounding culture media through constitutive overexpression of codon-optimized tyrosine ammonia lyase from *Trichosporon cutaneum* (*TAL*), feedback-inhibition-resistant 3-deoxy-d-arabinoheptulosonate-7-phosphate synthase (*aroG*^*fbr*^) and chorismate mutase/prephenate dehydrogenase (*tyrA*^*fbr*^) in a transcriptional regulator *tyrR* knockout strain. Next, to enhance the precursor malonyl-CoA supply, an inactivation-resistant version of acetyl-CoA carboxylase (*ACC1*^*S659A,S1157A*^) was introduced into the downstream *Saccharomyces cerevisiae* module constitutively expressing codon-optimized 4-coumarate-CoA ligase from *Arabidopsis thaliana* (*4CL*) and resveratrol synthase from *Vitis vinifera* (*STS*), and thus further improve the conversion of *p*-coumaric acid-to-resveratrol. Upon optimization of the initial inoculation ratio of two populations, fermentation temperature, and culture time, this co-culture system yielded 28.5 mg/L resveratrol from glucose in flasks. In further optimization by increasing initial net cells density at a test tube scale, a final resveratrol titer of 36 mg/L was achieved.

**Conclusions:**

This is first study that demonstrates the use of a synthetic *E. coli*–*S. cerevisiae* consortium for de novo resveratrol biosynthesis, which highlights its potential for production of other *p*-coumaric-acid or resveratrol derived biochemicals.

## Background

**Fig. 1:**
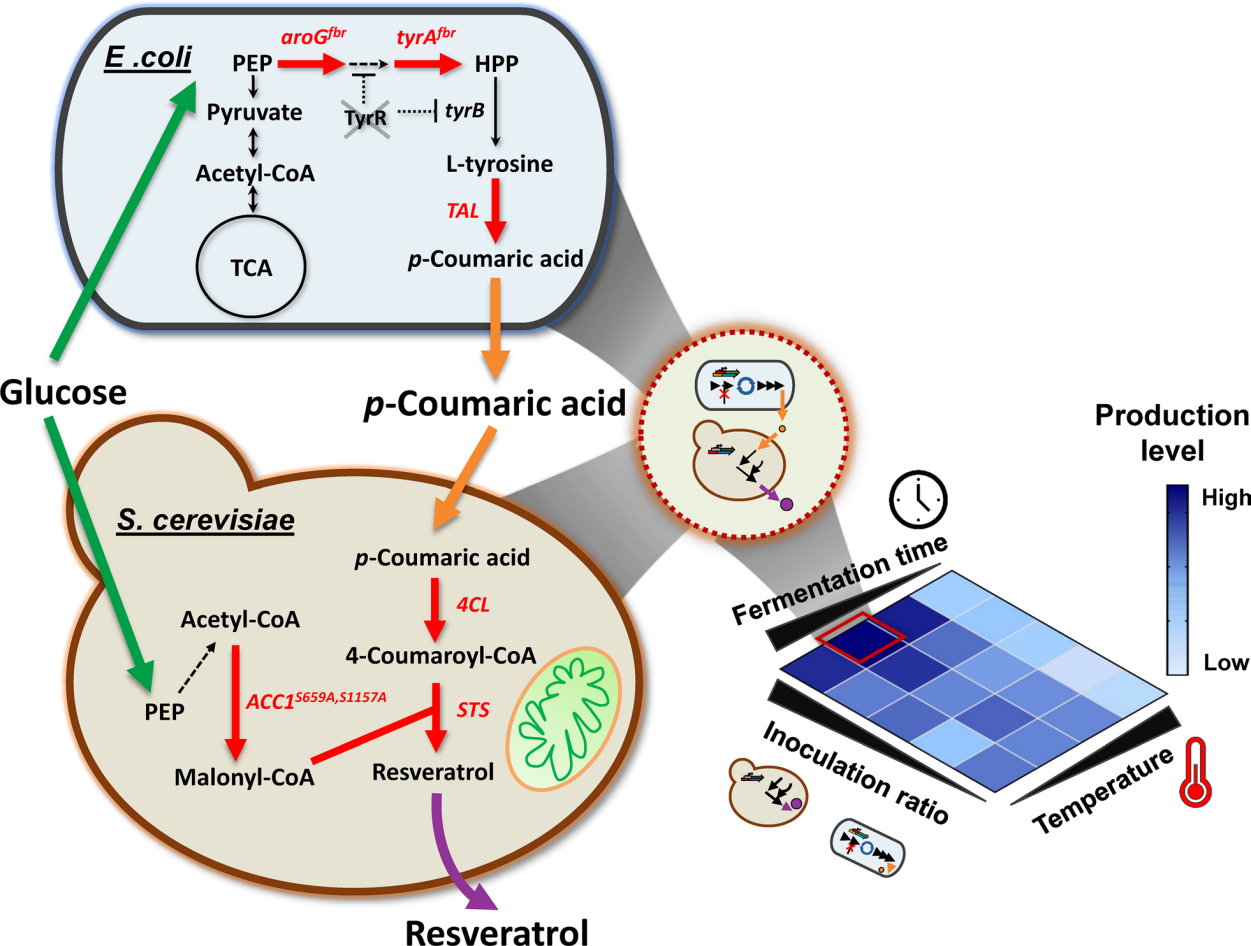
Overview of the *E. coli*–*S. cerevisiae* co-culture system for de novo resveratrol biosynthesis. The resveratrol pathway is divided into two modules for co-culture-based biosynthesis: the upstream *E. coli* module for *p*-coumaric acid production and the downstream *S. cerevisiae* module for *p*-coumaric acid-to-resveratrol conversion. Improved resveratrol production can be achieved through optimization of inoculated cell number ratios, fermentation temperatures, and cultivation times. A solid line represents an enzymatic reaction through an indicated enzyme whereas the dashed line represents reaction involving multiple enzymes. Overexpressed enzymes are labeled in red text with red arrows. Enzymes encoded by the genes shown are *aroG*^*fbr*^, feedback-inhibition-resistant 3-deoxy-d-arabinoheptulosonate-7-phosphate synthase; *tyrA*^*fbr*^, feedback-inhibition-resistant chorismate mutase/prephenate dehydrogenase; *TAL*, tyrosine ammonia lyase; *4CL*, 4-coumarate-CoA ligase; *STS*, resveratrol synthase; *ACC1*^*S659A,S1157A*^, inhibition-resistant acetyl-CoA carboxylase. The *tyrR*, a transcription factor that represses tyrosine synthesis pathway genes, is deleted in the upstream module

Resveratrol is a plant-derived stilbenoid compound, commonly found in grape extract and red wine, that is touted for bioactive properties including antioxidant, anti-inflammatory, anti-tumor, cardio- and neuro-protective properties [1–4]. Given the increasing interest in these health-related benefits, the global market for resveratrol is expected to almost double in the next 6 years from US$ 58 million (in 2020) to US$ 99.4 million by 2026 [5]. To meet this growing demand and bypass eco-unfriendly chemical syntheses and direct extraction from natural sources [6–8], there have been numerous metabolic engineering approaches for microbial resveratrol production [9–14]. Biochemically, resveratrol synthesis requires 4-coumaroyl-CoA whose biosynthesis starts with the conversion of phenylalanine and tyrosine into the phenylpropanoid acids cinnamic acid and *p*-coumaric acid, respectively [15]. These reactions are catalyzed by phenylalanine ammonia lyase (PAL) and tyrosine ammonia lyase (TAL) enzymes with some promiscuous cross-reactivity known to be present [16]. Cinnamic acid can be further hydroxylated by a cytochrome P-450-dependent cinnamate-4-hydroxylase (C4H) to form *p*-coumaric acid. In both routes, the resulting *p*-coumaric acid is subsequently biotransformed to 4-coumaroyl-CoA by 4-coumaroyl-CoA ligase (4CL) and then finally into resveratrol by the sequential condensations with malonyl-CoA catalyzed by a stilbene synthase (STS) [17] (Fig. 1).

Using the approaches of metabolic engineering, common host microorganisms including *E. coli* and *S. cerevisiae* as well as a variety of non-conventional hosts have been extensively engineered for resveratrol bioproduction [9–14, 18–20]. However, most efforts do not describe purely de novo production and thus require the supplementation of relatively expensive and low-water-solubility substrates such as *p*-coumaric acid or aromatic amino acids [15, 17]. One standout report for de novo production from glucose and ethanol was achieved in *S. cerevisiae* CEN.PK102-5B [10, 11] whereby extensive engineering of the tyrosine pathway along with complementation of resveratrol biosynthetic genes (*TAL* from *H. aurantiacus*, *4CL* from *A. thaliana* and *STS* from *V. vinifera*) led to a resveratrol titer of 416 and 531 mg/L from glucose and ethanol, respectively, in fed-batch fermentation [10]. Further improvements were made by using the phenylalanine pathway to achieve a final titer of 812 and 755 mg/L resveratrol from glucose and ethanol feed, respectively, in fed-batch mode [11].

Despite these decent titers, *S. cerevisiae* does not have a very strong innate flux toward aromatic amino acids and derived products. In circumstances wherein metabolic potential is restricted, co-culture strategies have been explored. In this regard, co-culture strategies can improve production by dividing complex and extensive pathways into individual modules, thus reducing the metabolic burden of each independent microbial strain and leveraging the innate metabolic potential of each host [21–24]. In doing so, this strategy enables parallel construction of optimized metabolic pathways and utilizes cross-feeding at key metabolite nodes [25, 26].

To date, there are only two published studies utilizing microbial co-culture for the production of resveratrol. The first case demonstrated an *E. coli*–*E. coli* co-culture using W3110s to produce resveratrol from glycerol [27]. In this scheme, the first *E. coli* module was engineered to produce *p*-coumaric acid through the overexpression of *TAL* from *Rhodothorula glutinis*, *aroG*^*fbr*^, and *tktA* in the background of a pheA knockout mutant. The second *E. coli* module utilized the *p*-coumaric acid and converted it into resveratrol via overexpression of heterologous genes *4CL* from *Streptomyces coelicolor* A2 and *STS* from *Vitis vinifera*. The resulting co-culture system led to a final titer of 22.6 mg/L resveratrol in a bioreactor while still requiring IPTG induction. In the second case, another *E. coli*–*E. coli* co-culture (this time using MG1655 strain background) produced 55.7 mg/L resveratrol from glucose [14]. In this scheme, the *p*-coumaric acid-producing strain was generated through the introduction of *aroG*^*fbr*^, *tyrA*^*fbr*^ and *R. glutinis TAL* into a *tyrR* and *pgi* (encoding the first-step enzyme of the EMP pathway) knockout background. The second strain produced resveratrol through heterologous overexpression of *C. glutamicum acc*, *Petroselinum crispum 4CL* and *Arachis hypogaea STS* in conjunction with a *zwf* deletion. As with the first case, this co-culture leveraged the P_Lteto-1_ promoter and thus requires induction by an expensive inducer such as doxycycline.

Based on these prior results, no study has used a co-culture system for resveratrol production without the need for expensive inducers and with distinct organisms. The only instances described above used an *E. coli*–*E. coli* co-culture strategy that does not leverage distinct metabolic capacities. In this work, we developed a unique consortium utilizing two metabolically distinct microorganisms, *E. coli* and *S. cerevisiae*, for de novo resveratrol production from glucose. In doing so, we utilize a direct, one-step route for conversion of tyrosine into *p*-coumaric acid through heterologous overexpression of a tyrosine ammonia lyase from *T. cutaneum* (*TAL*) in a *E. coli* tyrosine overproducer [28] (designated as the upstream module). In the second host, we chose *S. cerevisiae* to better express plant-derived resveratrol biosynthetic enzymes due to its ability for proper protein folding and post-translational modification. In this regard, we rewired this host to convert *p*-coumaric acid into resveratrol via chromosomally integrated expression of *ACC1*^*S659A,S1157A*^, *A. thaliana 4CL* and *V. vinifera STS* (designated as the downstream module). Through a series of optimization for media composition, inoculation ratios, fermentation temperatures, and initial net cells density, we obtained 36 mg/L resveratrol in a purely de novo fashion without the need for supplementation of expensive inducers or precursors. The platform described here thus enables the first demonstration of a synthetic *E. coli*–*S. cerevisiae* consortium for de novo resveratrol production.

## Results and discussion

### *Escherichia coli*–*S. cerevisiae* co-culture design and construction

In this work, we chose to select an *E. coli*–*S. cerevisiae* co-culture to take advantage of these two distinct organisms. As stated above, the downstream enzymes in this pathway are more compatible with the eukaryotic environment of *S. cerevisiae*. Additionally, previous reports have demonstrated that 4-coumaroyl-CoA can inhibit the activity of the upstream TAL enzyme [29]. As a result, separating the expression of TAL and 4CL enzymes would bypass an undesired feedback-inhibitory crosstalk within the same host. The basic design for this synthetic co-culture is shown in Fig. 1.

We constructed the upstream module in *E. coli* by taking advantage of a more robust metabolic potential for aromatic amino acid pathways. To do so, we created a tyrosine overproducer strain of *E. coli* BL21(DE3) consisting of a *tyrR* knockout along with constitutive overexpression of feedback-inhibition-resistant versions of *aroG*^*fbr*^ and *tyrA*^*fbr*^ [28]. In this background, we then redirected metabolic flow from intracellular tyrosine pools to *p*-coumaric acid by expressing a heterologous, codon-optimized *T. cutaneum TAL* gene (Additional file 1: Table S2) [30] under the control of a constitutive promoter with a strong ribosomal binding site. The resulting strain (named eBL0430T) exhibited a high titer of *p*-coumaric acid (414 mg/L) with good biomass production, especially compared to a strain with lower gene expression and production level (named strain eBL0432T producing 122 mg/L, Additional file 1: Fig. S1). As a result, this strain was selected for use in the co-culture.

To establish the downstream module for *p*-coumaric acid-to-resveratrol conversion in *S. cerevisiae*, we chromosomally integrated constitutive expression cassettes for codon-optimized *4CL* and *STS* (Additional file 1: Table S2) [10] into the BY4741 strain. To increase the supply of intracellular malonyl-CoA, we subsequently integrated a feedback resistant mutant, *ACC1*^*S659A,S1157A*^ [10], into this strain to yield a final strain (named sBY11). This resulting strain exhibited bioconversion of *p*-coumaric acid into resveratrol and thus was selected for use in the co-culture.

**Fig. 2:**
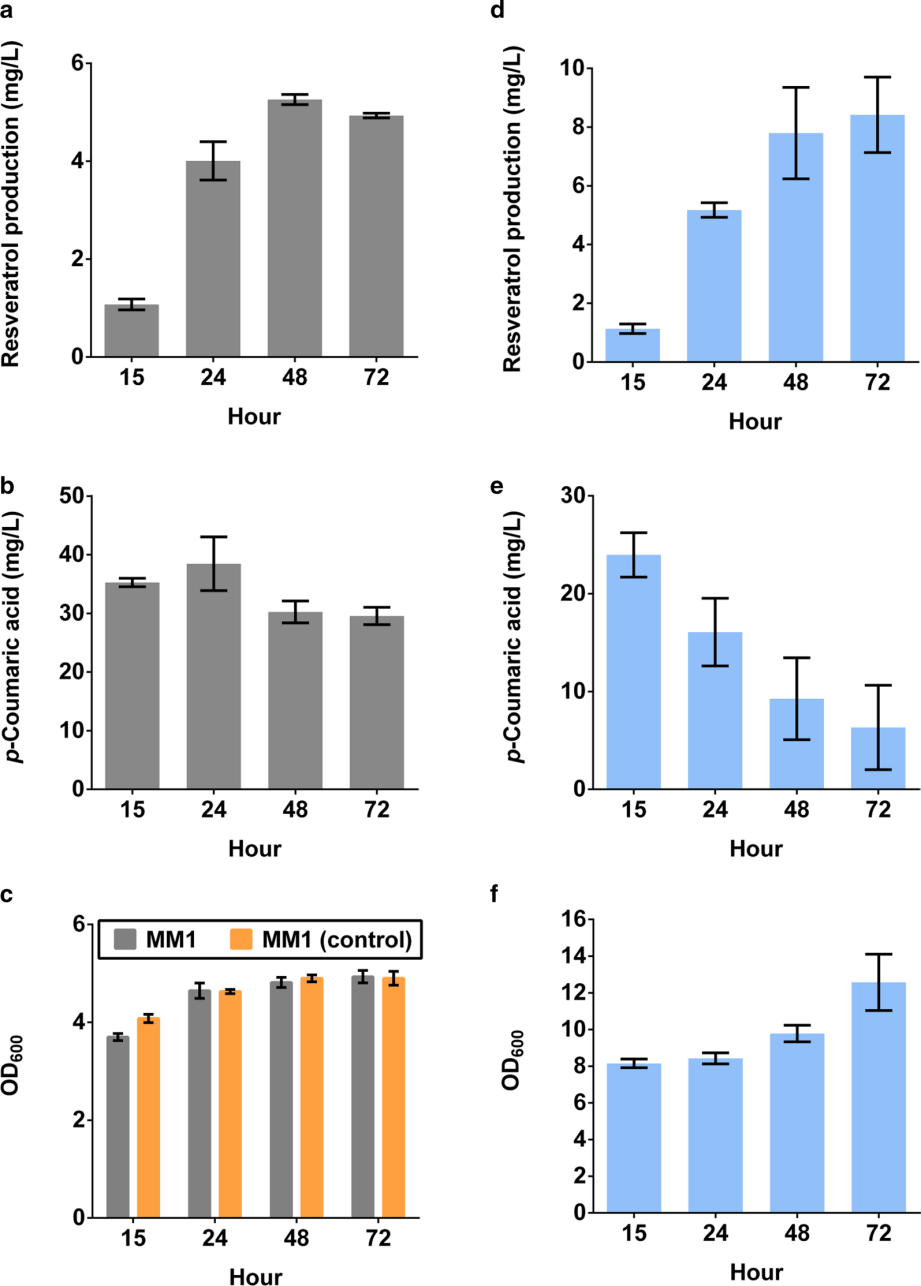
*De novo* resveratrol production from glucose using the synthetic consortia. Comparisons of the consortia performance in **a–c** minimal media MM1, and **d–f** complex media RM1. Time-course profiles of **a**, **d** resveratrol production, **b**, **e** accumulated *p*-coumaric acid as well as **c**, **f** biomass formation. All media contain 20 g/L glucose. **c** The growth status of a non-*p*-coumaric acid producer *E. coli* eBL0400DT-yeast sBY11 consortium was used as a control. The experiments were conducted with inoculation ratio of 1:1 and initial net cells density of 3 × 10^6^ cells per mL of culture. Each data point and error bars represent means and standard deviations from biological triplicates, respectively

Once these two hosts were constructed, we evaluated the synthetic co-culture’s capacity to product resveratrol in a de novo manner. Specifically, we tested production in a minimal medium (MM1) using an inoculation ratio of 1:1 with a middle-ground co-cultivated temperature of 33.5 °C (Fig. 2a–c). In this condition, a maximum resveratrol titer of 5.3 mg/L was achieved at 48-h timepoint (with a yield of 0.26 mg resveratrol/g glucose) (Fig. 2a), however, a higher amount of *p*-coumaric acid (30.2 mg/L) was observed in this condition (Fig. 2b). Moreover, the growth of this co-culture (Fig. 2c) indicated that the conversion issues could be due to the poor co-culture growth in this minimal media formulation.

Previous studies have demonstrated significantly improved consortia performance and biomass formation with the addition of some nutrients. As examples, increasing the concentration of yeast extract from 1 g/L to 2 g/L in an *E. coli*–*E. coli* co-culture resulted in a nearly 136-fold increase in monolignol *p*-coumaryl alcohol production [24]. Additionally, nutrient optimization in an *E. coli*–*S. cerevisiae* consortium led to a 3.1-fold increase in naringenin biosynthesis [31]. With these results as context, we investigated a nutrient-rich media formulation (RM1) to test its effect on co-culture performance (Fig. 2d–f). In doing so, we repeated the *E. coli* eBL0430T–*S. cerevisiae* sBY11 co-culture at 33.5 °C with an inoculation ratio of 1:1. In this case, the consortia was able to produce more resveratrol (7.8 mg/L vs. 5.3 mg/L) and accumulated less *p*-coumaric acid (9.3 mg/L vs. 30.2 mg/L) when compared with that of MM1 medium used above (compare Fig. 2d, e with Fig. 2a, b). Moreover, under this culture condition, resveratrol was gradually produced over time with concomitant decrease in *p*-coumaric acid, thus implying that the downstream yeast module was more apt to convert this substrate in this media condition. Furthermore, biomass accumulation was enhanced in this complex RM1 medium compared with the defined medium above (comparing Fig. 2f to c). As a result, the RM1 medium was used for the following experiments.

### Investigating the impacts of inoculation ratio and fermentation temperature on resveratrol biosynthesis

**Fig. 3:**
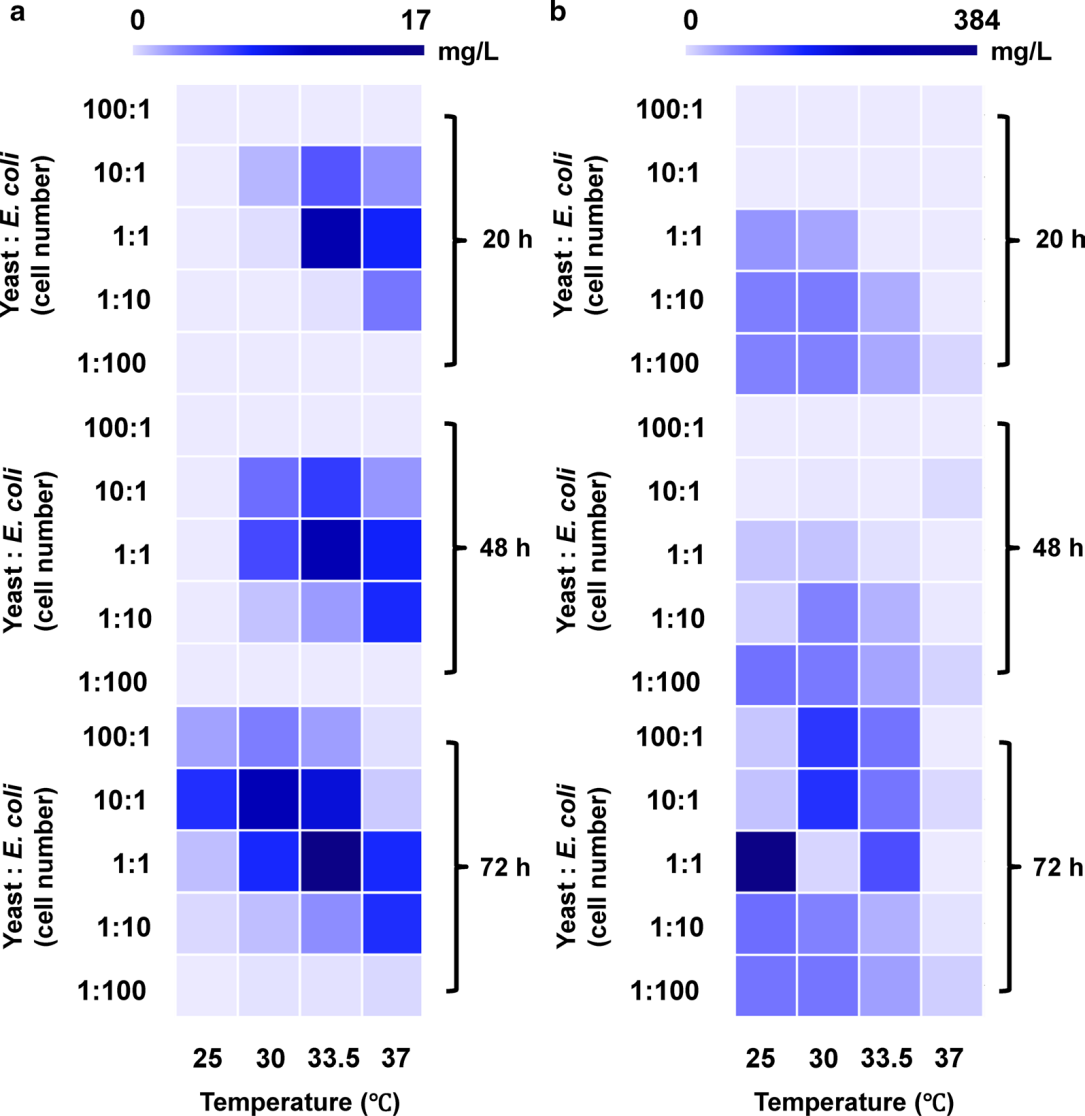
Investigating the impact of fermentation conditions on resveratrol production with the use of test-tube screening. Heat maps for consortia performance of **a** resveratrol production and **b***p*-coumaric acid accumulation using co-cultures with altered inoculation cell number ratios, fermentation temperatures, and culture times. Experiments were conducted with a constant initial net cell density of 3 × 10^6^ cells per mL. The color scale bar shown represents the concentration of indicated metabolites. Data points are mean values with n = 3 biological replicates. Bar graphs containing this data with error bars can be found in the Additional file 1: Fig. S3

Maintaining a stable and robust composition of organisms within a co-culture is essential for efficient biochemical production [26]. In this case, we are utilizing two organisms with different optimal temperatures for growth thus implying culture temperature as an important parameter in co-culture performance. To this end, we explored the impacts of varying fermentation temperature (25, 30, 33.5 and 37 °C), time (20, 48, and 72 h), and initial inoculation ratio of engineered strains (100:1, 10:1, 1:1, 1:10 to 1:100) in a large-scale test tube system (Fig. 3 and Additional file 1: Fig. S3).

In general, cultivation at the two higher temperatures (33.5 and 37 °C) displayed higher productivities and titers of resveratrol during the early-middle stage of fermentation (20 and 48 h) compared with the lower temperature range of 25–30 °C (Table 1 and Fig. 3a). Specifically, the synthetic consortia incubated at these high temperatures (33.5–37 °C) exhibited averaged resveratrol titers that were up to 15.74-fold higher than those at lower temperatures (Additional file 1: Fig. S3a, b). Additionally, these elevated temperature cultures also produced nearly 4-fold less *p*-coumaric acid than that at relatively low temperatures (25 and 30 °C) (Fig. 3b and Additional file 1: Fig. S3d, e). These results demonstrated that a higher cultivation temperature range of 33.5–37 °C resulted in improved resveratrol productivity from the consortia at the early-to-middle phase of fermentation, thus leading to a faster conversion of *p*-coumaric acid into resveratrol.

**Table 1:** Comparison of the effects of inoculation ratio and fermentation temperature on resveratrol productivity of the synthetic co-culture at a test tube scale with a constant initial net cell density (3 × 10^6^ cells per mL of culture)

Fermentation time (hour)	Inoculation ratio (Yeast: *E. coli*)	Resveratrol productivity (mg/L/h)			
		25 °C	30 °C	33.5 °C	37 °C
20	100:1	0.00 ± 0.00	0.00 ± 0.00	0.00 ± 0.00	0.00 ± 0.00
	10:1	0.00 ± 0.00	0.08 ± 0.03	0.24 ± 0.08	0.13 ± 0.04
	1:1	0.00 ± 0.00	0.02 ± 0.01	0.57 ± 0.04^a^	0.35 ± 0.02
	1:10	0.00 ± 0.00	0.00 ± 0.00	0.01 ± 0.02	0.18 ± 0.02
	1:100	0.00 ± 0.00	0.00 ± 0.00	0.00 ± 0.00	0.00 ± 0.00
48	100:1	0.00 ± 0.00	0.00 ± 0.00	0.00 ± 0.00	0.00 ± 0.00
	10:1	0.00 ± 0.00	0.08 ± 0.01	0.12 ± 0.03	0.05 ± 0.01
	1:1	0.00 ± 0.00	0.11 ± 0.01	0.23 ± 0.02	0.15 ± 0.01
	1:10	0.00 ± 0.00	0.02 ± 0.00	0.05 ± 0.00	0.14 ± 0.02
	1:100	0.00 ± 0.00	0.00 ± 0.00	0.00 ± 0.00	0.00 ± 0.00
72	100:1	0.03 ± 0.01	0.05 ± 0.01	0.03 ± 0.01	0.00 ± 0.00
	10:1	0.09 ± 0.02	0.15 ± 0.05	0.12 ± 0.05	0.01 ± 0.01
	1:1	0.02 ± 0.00	0.09 ± 0.02	0.23 ± 0.02	0.09 ± 0.01
	1:10	0.01 ± 0.01	0.02 ± 0.01	0.04 ± 0.02	0.09 ± 0.00
	1:100	0.00 ± 0.00	0.00 ± 0.00	0.00 ± 0.00	0.01 ± 0.00

^a^Denotes that the condition with a maximum productivity of resveratrol

Despite these results at early-to-middle range, the averaged final titer of the conditions incubated at 37 °C across a range of inoculation ratio (100:1–1:100; with 3.01 mg/L resveratrol) was lower than that of 30 °C (4.42 mg/L) and 33.5 °C (6.08 mg/L). This result indicates that the consortia’s metabolic activity at 37 °C suffered in the later phase of fermentation (48–72 h) compared with the lower temperatures, suggesting 33.5 °C was a more favorable fermentation temperature for the synthetic consortia when operating in batch culture mode.

As expected, the final resveratrol content was significantly influenced by the inoculation ratio (tested from 100:1 to 1:100) across a range of temperatures (25–37 °C) and over time (20–72 h) (Fig. 3a and Additional file 1: Fig. S3a–c). The averaged resveratrol titers of conditions with higher inoculated yeast-to-*E. coli* ratios (100:1 and 10:1) were between 1.21 and 7.70-fold higher than the conditions with lower inoculation ratios (1:10 and 1:100) (Fig. 3a). These results highlight that the downstream yeast strain was the rate limiting module for *p*-coumaric acid-to-resveratrol conversion, especially when operating the synthetic co-culture platform at a temperature range of 25–33.5 °C. Among all the conditions, the inoculation ratio of 1:1 exhibited the highest averaged final resveratrol titer of 7.83 mg/L (Fig. 3a and Additional file 1: Fig. S3a–c), thus indicating that the 1:1 ratio was the optimal inoculation ratio for the synthetic consortia.

Using the information generated in this analysis, we were able to achieve a maximum resveratrol productivity of 0.57 mg/L/h when fermentation was conducted with an inoculation ratio of 1:1 at 33.5 °C (Table 1). Specifically, a maximum resveratrol titer of 16.6 mg/L was obtained along with 120.16 mg/L *p*-coumaric acid accumulated at the end of the 72 h fermentation (Fig. 3a, b and Additional file 1: Fig. S3c, f). As a result, these conditions were used for a flask-scale up as discussed in the next section.

### Scale up resveratrol production using the synthetic consortia at a shake flask scale

**Fig. 4:**
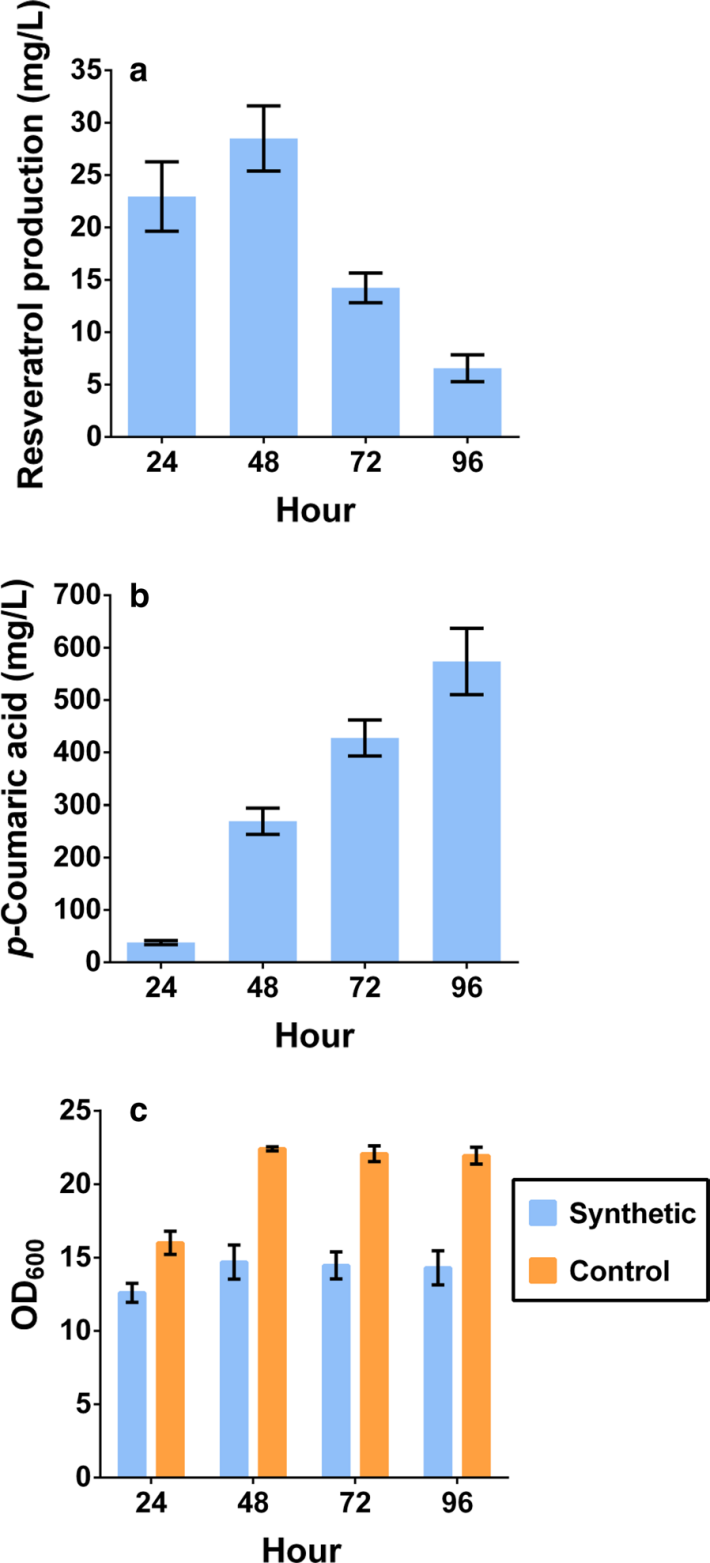
Evaluation of the synthetic co-culture capacity for resveratrol production in flasks. Comparisons of **a** resveratrol production, **b** accumulated *p*-coumaric acid and **c** growth profile (labeled as Synthetic) for the designed *E. coli* eBL0430T-yeast sBY11 co-culture at 33.5 °C are shown. **c** The growth dynamics for a non-*p*-coumaric acid producer *E. coli* eBL0400DT-yeast sBY11 consortium (labeled as Control) were similarly cultivated at 33.5 °C to be used as a control. The experiments were conducted with constant inoculation ratio of 1:1 and initial net cells density of 3 × 10^6^ cells per mL of culture. Each data point and error bar represent means and standard deviations from biological triplicates, respectively

Process scale-up is an important aspect for industrial biofuel or biochemical production [32, 33]. Based on the optimal condition achieved at the test tube scale (16.6 mg/L at 72-h timepoint using a 1:1 inoculation ratio with 33.5 °C temperature), we sought to evaluate the scalability of this synthetic co-culture at a shake flask scale with an extended fermentation period (96 h). To this end, we measured resveratrol production, accumulated *p*-coumaric acid level, and co-culture growth profile across more timepoints (Fig. 4 and Table 2). In this condition, we found a 1.68-fold improvement at flask scale resulting in a maximum resveratrol productivity of 0.96 mg/L/h (Fig. 4a and Table 2). Additionally, a maximum resveratrol titer of 28.5 mg/L was achieved at the flask scale (Fig. 4a), which was 1.72-fold higher than that at the test tube scale. This result implied that the synthetic consortium was more metabolically active for resveratrol production at the flask scale than that at the test tube scale, possibly due to better aeration, mass transfer and agitation provided in a shake flask [34, 35]. However, resveratrol gradually decreased after 48 h accompanied by an increase in *p*-coumaric acid accumulation (Fig. 4a, b). Furthermore, a relatively lower biomass formation was seen in this engineered *p*-coumaric acid *E. coli* eBL0430T-yeast sBY11 co-culture compared to a control co-culture containing non-*p*-coumaric acid producer *E. coli* eBL0400DT and engineered *S. cerevisiae* sBY11 strains (Fig. 4c). These results highlight a potential challenge with yeast as a production host in that resveratrol exhibits better antifungal (with minimum inhibitory concentrations (MICs) of 10–20 µg/mL for *S. cerevisiae*) than antibacterial activity (with MICs of 57–1000 µg/mL for *E. coli* depending on species) [36, 37]. As a result, we tested higher inoculum sizes to prevent growth being influenced by this molecule.

**Table 2:** Resveratrol productivity of the synthetic co-culture at a shake flask scale with inoculation ratio (1:1), initial net cells density (3 × 10^6^ cells per mL of culture), and 33.5 °C temperature

Fermentation time	Resveratrol productivity (mg/L/h)
24 h	0.96 ± 0.14^a^
48 h	0.59 ± 0.07
72 h	0.20 ± 0.02
96 h	0.07 ± 0.01

^a^Denotes that the condition with a maximum productivity of resveratrol

### Increased net inoculum size of the co-culture improves resveratrol production

**Fig. 5:**
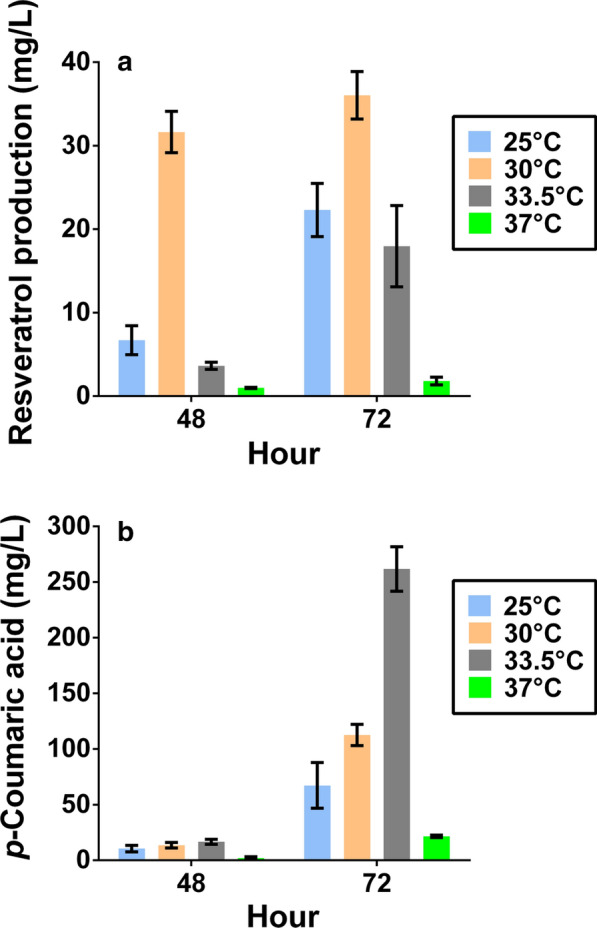
Investigating the impact of increasing initial net inoculation cell density on co-culture performance. Comparisons of **a** resveratrol production and **b** accumulated *p*-coumaric acid for the synthetic co-culture was conducted at various temperatures (25–37 °C). The experiments were conducted with a constant inoculation ratio of 1:1 while changing the initial net cells density to 3 × 10^7^ cells per mL of culture. Each data point and error bar represent means and standard deviations from biological triplicates, respectively

Previous studies have shown that utilizing high cell density microbial bioprocesses can increase volumetric productivity and alleviate the impact of toxic growth inhibitors [32, 38, 39]. Similarly, increasing co-culture inoculum size of *S. cerevisiae* and *E. coli* led to an improvement in naringenin production [31]. Therefore, we chose to investigate whether maintaining the same optimal inoculation ratio describe above (1:1) with a tenfold higher net cells density (namely from 3 × 10^6^ to 3 × 10^7^ cells per mL of culture) could enhance resveratrol production. To comprehensively investigate the impact of increasing inoculum size on consortia performance, we finally performed the fermentations at a range of temperature 25–37 °C in a test tube scale and measured resveratrol production as well as accumulated *p*-coumaric acid level (Fig. 5).

Interestingly, at these higher inoculum sizes, an increased maximum resveratrol productivity of 0.63 mg/L/h was achieved at 30 °C (Table 3) compared to the lower-density test tube experiment where the maximum was at 33.5 °C (Table 1). Furthermore, the co-culture seeded at a higher initial net cells density yielded a maximum resveratrol titer of 36.0 mg/L at 30 °C (Fig. 5a), a value that was nearly 5.35-fold higher than the lower cell density condition (6.74 mg/L) (Fig. 3a and Additional file 1: Fig. S3c). It should be noted that only 18.0 mg/L resveratrol was produced at 33.5 °C, the optimal temperature identified at a lower cell density seeding (Fig. 3a). Moreover, less accumulation of *p*-coumaric acid was observed (112.5 mg/L) when the fermentation was performed at 30 °C for 72 h when compared with the 33.5 °C condition (261.8 mg/L) (Fig. 5b). Additionally, the averaged resveratrol titer of the conditions conducted at the lower temperature range 25–33.5 °C for 72 h (25.4 mg/L) (Fig. 5a) was nearly 3.1-fold higher than the corresponding lower initial cell density conditions (8.2 mg/L) (Additional file 1: Fig. S3c). These results seem to indicate that the downstream yeast module was more metabolically active for conversion of *p*-coumaric acid into the end product at 30 °C than that at 33.5 °C when using high cell density fermentation strategy. As a result, this discrepancy in temperature optimum likely results from changes in the end-point population dynamics and metabolic potential caused by differences in initial starting density.

**Table 3:** Comparison of the effects of fermentation temperature on resveratrol productivity of the synthetic co-culture at a test tube scale with a constant inoculation ratio (1:1) and initial net cell density (3 × 10^7^ cells per mL of culture)

Fermentation time	Resveratrol productivity (mg/L/h)			
	25 °C	30 °C	33.5 °C	37 °C
48 h	0.14 ± 0.04	0.63 ± 0.06^a^	0.08 ± 0.01	0.02 ± 0.00
72 h	0.31 ± 0.04	0.50 ± 0.04	0.25 ± 0.07	0.03 ± 0.01

^a^Denotes that the condition with a maximum productivity of resveratrol

## Conclusions

In this study, we first establish a novel *E. coli*–*S. cerevisiae* consortium platform for de novo resveratrol biosynthesis from glucose via modular co-culture engineering. Through optimization of fermentation parameters, including altering inoculation ratios of co-culture, cultivation temperatures and times, we found that the downstream yeast module is a rate limiting node for *p*-coumaric acid-to-resveratrol conversion. This unique consortium enables resveratrol production with a high titer of 28.5 mg/L at a shake flask scale that can be improved to 36 mg/L resveratrol in a test tube when using higher cell density inoculations. While this work was conducted at a small scale, they nevertheless point toward advances in the use of co-cultures and exceed the value of another *E. coli*-*S. cerevisiae* co-culture for a similar polyketide, naringenin (obtaining 21 mg/L) [31]. These findings advance complex natural product biosynthesis with the use of bacterium-yeast co-culture approach. Several genetic strategies could further enhance resveratrol production using our *E. coli*-*S. cerevisiae* co-culture scenario, including (i) driving more metabolic flux from precursor *p*-coumaric acid toward resveratrol biosynthesis through multicopy integration of resveratrol biosynthetic genes *4CL* and *STS* into long-terminal repeat (LTR)-retrotransposons of *S. cerevisiae* such as Ty1 or Ty4 elements [10, 40], (ii) scaling up the fermentation in a fed-batch controlled mode via monitoring co-culture growth status and constantly feeding low level of glucose to avoid overflow metabolism (i.e. formation of side metabolites such as acetate for *E. coli* and ethanol for *S. cerevisiae*), and (iii) executing more sophisticated genetic manipulations for creating a mutualistic consortium to further reduce metabolite inhibitions on consortia growth and thus improve the final yield (i.e. *S. cerevisiae* can only utilize acetate excreted by *E. coli* when using xylose as a carbon source without producing ethanol [41], or rewiring metabolism of *S. cerevisiae* to render a Crabtree-negative yeast [42]). Finally, on a molecular side, addressing limited bioavailability of this molecule by attaching sugar moieties to resveratrol can improve stability and solubility in aqueous solutions [43, 44]. Nevertheless, these results demonstrate the viability of a co-culture approach for production of resveratrol.

## Materials and methods

### Strains, media and plasmid or integrative expression cassette construction

All strains and plasmids used in this study are listed in Table 4. Sequences of primers synthesized by Integrated DNA Technologies (Coralville, IA) and codon-optimized DNA fragments used in this work are listed in Additional file 1: Tables S1 and S2, respectively. All Gibson-assembled DNA [45] were electroporated (2 mm Electroporation Cuvettes, Bioexpress) into *E. coli* competent cells with a BioRad Genepulser Xcell at 2.5 kV. *E. coli* NEB10β was used for gene cloning or propagation of all expression vectors. It was cultivated in Luria–Bertani (LB) medium (1% tryptone, 0.5% yeast extract and 1% NaCl) supplemented with appropriate antibiotics (50 μg/mL kanamycin or 50 μg/mL spectinomycin (Sigma)) with 225 rpm orbital shaking at 37 °C. The Frozen EZ Yeast Transformation II Kit (Zymo Research) was used to transform an integrative expression cassette into the yeast, and the resulting yeast transformants were selected on yeast synthetic complete (YSC) media with the appropriate dropouts for auxotrophic selection. RM1 (1X CSM-URA-LEU (MP Biomedicals), 1X Yeast Nitrogen Base (BD Difco) at 5 g/L of ammonium sulfate, 1% tryptone, 0.5% yeast extract, 1% NaCl, 20 g/L d-glucose, 50 μg/mL kanamycin and 50 µg/mL spectinomycin) and MM1 (1X CSM-URA-LEU (MP Biomedicals), 1X Yeast Nitrogen Base (BD Difco) at 5 g/L of ammonium sulfate, 1X M9 minimal salts (Sigma), 2 mM MgSO_4_, 0.1 mM CaCl_2_, 20 g/L d-glucose, 50 μg/mL kanamycin and 50 µg/mL spectinomycin) were used for medium optimization studies.

**Table 4:** List of strains and plasmids used in this study

Strain/plasmid	Description	Source
*E. coli* strain		
NEB10β	Δ*(ara*-*leu) 7697 araD139 fhuA* Δ*lacX74 galK16 galE15 e14*- *ϕ80dlacZΔM15 recA1 relA1 endA1 nupG rpsL (Str*^*R*^*) rph spoT1* Δ*(mrr*-*hsdRMS*-*mcrBC)*	New England Biolabs
BL21(DE3)	*E. coli* str. B F^−^ *ompT* *gal* *dcm* *lon* *hsdS*_*B*_(*r*_*B*_^−^*m*_*B*_^−^) *λ(DE3 [lacI* *lacUV5*-*T7p07* *ind1* *sam7* *nin5*]) [*malB*^+^]_K-12_(λ^S^)	New England Biolabs
eBL04	[BL21(DE3)] Δt*yrR* pET28-pYIBN-*aroG*^(fbr)^-B30rbs-*tyrA*^(fbr)^-tRRNC; Kan^R^	[28]
eBL0400DT	[eBL04] pCDFDuet-1; Kan^R^; Spc^R^	[28]
eBL0430T	[eBL04] pCDF-pLPP-B30rbs-*TcXAL*-T7t; Kan^R^; Spc^R^	This study
eBL0432T	[eBL04] pCDF-pLPP-B32rbs-*TcXAL*-T7t; Kan^R^; Spc^R^	This study
*S. cerevisiae* strain		
BY4741	*MATα SUC2 gal2 mal2 mel flo1 flo8*-*1 hap1 ho bio1 bio6 his3*Δ1 *leu2*Δ0 *met15*Δ0 *ura3*Δ0	ATCC
sBY10	[BY4741] pTEF1-*At4CL*-tADH1-pPGK1-*VvSTS*-tCYC1 (integration with *Kluyveromyces lactis* LEU2 marker)	This study
sBY11	[sBY10] pTEF1-*ACC1*^*S659A,S1157A*^-tADH1 (integration with *K. lactis* LEU2 and URA3 markers)	This study
Plasmids		
pCDF-Duet-1	For construction *p*-coumaric acid producing plasmids	[28]
pETM-TAL-4CL	For amplification of *TcXAL* gene	[30]
pCfB1020	For construction resveratrol expression cassette	[10]
pCfB1175	For construction *ACC1* mutant expression cassette	[10]
pCDF-pLPP-B30rbs-*TcXAL*-T7t	For *p*-coumaric acid production testing	This study
pCDF-pLPP-B32rbs-*TcXAL*-T7t	For *p*-coumaric acid production testing	This study

For construction of pCDF-pLPP-B30rbs-TcXAL-T7t, Gibson assembly method was employed to combine an amplicon containing *TAL* gene amplified from pETM-TAL-4CL [30] with primers P80 as well as P81, primer P67, and the PCR product amplified from pCDF-Duet-1 empty vector [28] with primers 82 and 83. To construct pCDF-pLPP-B32rbs-TcXAL-T7t, the DNA fragment PCR-amplified from pETM-TAL-4CL [30] with primers P84 as well as P81, was Gibson assembled with the primer P68 and the amplicon amplified from pCDF-Duet-1 empty vector [28] with primers 82 as well as 83. To generate a yeast integrative expression cassette carrying *4CL* and *STS* genes, primers P85 and P86 were used for amplifying a linear DNA fragment from pCfB1020 [10]. To yield an integrative cassette containing post-translational deregulated *ACC1*^*S659A,S1157A*^ gene, primers P89 and P90 were used for amplifying a linear DNA fragment from pCfB1175 [10].

### Upstream *E. coli* module construction and *p*-coumaric acid production

To construct the upstream module of the design co-culture, the *p*-coumaric acid producing plasmids pCDF-pLPP-B30rbs-TcXAL-T7t and pCDF-pLPP-B32rbs-TcXAL-T7t were individually transformed into eBL04 [28] leading to eBL0430T and eBL0432T strains, respectively. The resulting strains were able to constitutively overexpress *T. cutaneum* TAL enzyme. For *p*-coumaric acid production, starter culture of *E. coli* was grown in 2 mL LB medium containing 50 μg/mL kanamycin and 50 μg/mL spectinomycin with 225 rpm orbital shaking at 30 °C overnight. Then seed culture was inoculated into 3 mL LB medium supplemented with antibiotics with an initial OD_600_ of 0.05 and incubated at 30 °C for 18 h. After fermentation, suspension culture was mixed with equal volume of absolute ethanol and centrifuged at 16,000*g* for 2 min. The supernatant fraction was collected for measurement of *p*-coumaric acid production using HPLC. The cell growth was measured by Ultrospec 2100 Pro UV/Visible Spectrophotometer observing optical density at 600 nm.

### Downstream yeast module construction and resveratrol production

To construct the downstream module of the design co-culture, we first integrated the constitutive expression cassette containing heterologous *4CL* and *STS* genes into *S. cerevisiae MCH2* locus located at chromosome XI [10]. The resulting transformants were selected on YSC dropout media (CSM-LEU) for auxotrophic selection and verified by colony PCR with primers P87 and P88. The resulting yeast strain, designated as sBY10, was subsequently transformed with an integrated expression cassette harboring *S. cerevisiae ACC1*^*S659A,S1157A*^ gene and homology arms targeting to an insertion site located between *NCA3* and *ASF1* loci at *S. cerevisiae* chromosome X [10]. The resulting transformants were selected on YSC dropout media (CSM-URA-LEU) and verified by colony PCR with primers P91 and P92, leading to a yeast strain sBY11. Prior to all fermentation tests, the starter *E. coli* and yeast cultures were inoculated from glycerol stocks into 3 mL LB (supplemented with 50 μg/mL kanamycin and 50 μg/mL spectinomycin) and YSC dropout media (CSM-URA-LEU), respectively. All seeding cultures were incubated at 30 °C.

For optimization of fermentation medium, *E. coli* eBL0430T was co-cultured with *S. cerevisiae* sBY11 and its capacity of resveratrol production was evaluated using RM1 or MM1 medium. The fermentation was performed with seeding the same initial cell density of 1.5 × 10^6^ cells/mL for each strain in test tubes containing 3 mL RM1 or MM1 medium. The cultures were afterwards incubated at 33.5 °C. A non-*p*-coumaric acid producer *E. coli* eBL0400DT [28] cocultured with yeast sBY11 strain was used as a control consortium (Fig. 2c and Additional file 1: Fig.S2). To investigate the impacts of inoculation ratios, fermentation temperatures and times on resveratrol production, various yeast: *E. coli* cell ratios (100:1, 10:1, 1:1, 1:10 and 1:100 with a constant initial net cells density of 3 × 10^6^ cells per mL of culture) as well as temperatures (25, 30, 33.5 and 37 °C) were adopted for production testing in test tubes containing 3 mL RM1 media. For evaluating the co-culture performance at a shake flask scale, fermentations were carried out at 33.5 °C using 25 mL RM1 media in 125 mL-flasks. All conditions were conducted at an inoculation ratio of 1:1 with a constant initial net cells density of 3 × 10^6^ cells per mL of culture. To investigate the effect of increasing initial net cells density on the consortia’s capacity for *p*-coumaric-to-resveratrol conversion, initial net coculture inoculum was increased from original 3 × 10^6^ to 3 × 10^7^ cells per mL of culture, and fermentations were performed at various temperatures (25, 30, 33.5 and 37 °C) while keeping the inoculation ratio constant at 1:1.

All timepoint samples were mixed with equal volume of absolute ethanol and centrifuged at 16,000 g for 2 min. The supernatants were used to analyze resveratrol and *p*-coumaric acid by HPLC.

### HPLC analysis

Samples from fermentations were filtered with 0.2-μm nylon syringe filters (Wheaton Science) prior to running HPLC. HPLC confirmation of resveratrol or *p*-coumaric acid production was performed using a Dionex UltiMate 3000 (Thermo Fisher Scientific) equipped with an Agilent Eclipse Plus C18 column (3.0 × 150 mm, 3.5 μm) with detection wavelength at 304 nm. Column oven was held at 25 °C with 1% acetic acid in water or acetonitrile as the mobile phase over the course of the 20-min sequence under the following conditions: 5% to 15% organic (vol/vol) for 5 min, 15% to 100% organic (vol/vol) for 8 min, 100% organic (vol/vol) for 2 min, 100% to 5% organic for 2 min followed by 5% organic for 3 min. The constant flow rate was set at 0.8 mL min-1. A standard curve was prepared using ≥ 99% purity resveratrol or ≥ 98.0% purity *p*-coumaric acid from Sigma-Aldrich.

## Supplementary information

**Additional file 1.**

## Data Availability

All data generated or analyzed in this study are included in the manuscript and its additional file.
