# Supplementary material for: De novo resveratrol production through modular engineering of an Escherichia coli–Saccharomyces cerevisiae co-culture
Source: Microb Cell Fact. 2020 Jul 14;19:143. doi: 10.1186/s12934-020-01401-5 (PMC7362445; doi:10.1186/s12934-020-01401-5)
Supplement: Supplementary file 1 — Additional file 1. [file 12934_2020_1401_MOESM1_ESM.pdf]

Additional file 1 for

De novo resveratrol production through modular co-culture engineering of an *Escherichia coli–Saccharomyces cerevisiae* co-culture

Shuo-Fu Yuan[1], Xiunan Yi[1], Trevor G. Johnston[2], and Hal S. Alper[1,3*]

[1]Institute for Cellular and Molecular Biology, The University of Texas at Austin, Austin, TX, USA

[2]Department of Chemistry, University of Washington, Box 351700, Seattle, WA, USA

[3]McKetta Department of Chemical Engineering, The University of Texas at Austin, Austin, TX, USA

[*] Corresponding author at: McKetta Department of Chemical Engineering, The University of Texas at Austin, 200 E Dean Keeton St. Stop C0400, Austin, TX 78712, United States. E-mail address: halper@che.utexas.edu (Hal S. Alper).

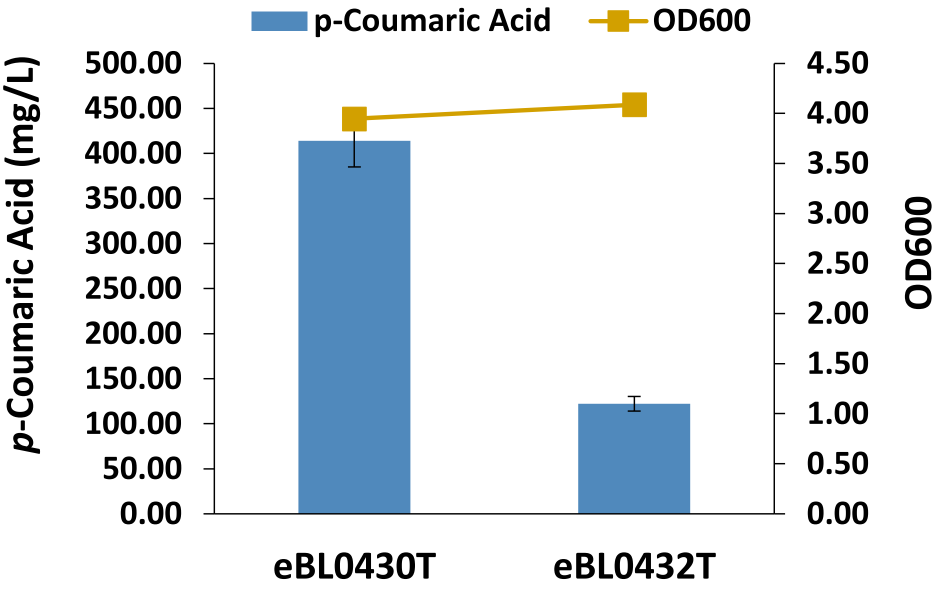

Fig. S1. Biomass measurement and *p*-coumaric acid production in *E. coli*. The comparison of biomass formation and *p*-coumaric acid production between engineered *E. coli* strains. The highest *p*-coumaric acid producer eBL0430T strain was selected for co-culture resveratrol production experiments. Each data point and error bar represent means and standard deviations from biological triplicates, respectively.

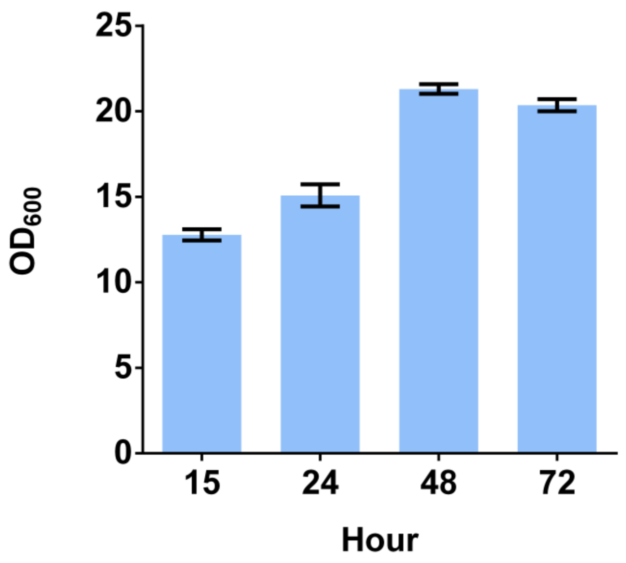

Fig. S2. Biomass formation of the control consortium cultivated in the RM1 medium. The growth status of a non-*p*-coumaric acid producer *E. coli* eBL0400DT-yeast sBY11 consortium grown at 33.5 °C in the complex RM1 medium is shown. The experiments were conducted with inoculation ratio of 1:1 and initial net cells density of 3 x 10^6 cells per mL of culture. Each data point and error bars represent means and standard deviations from biological triplicates, respectively.

a

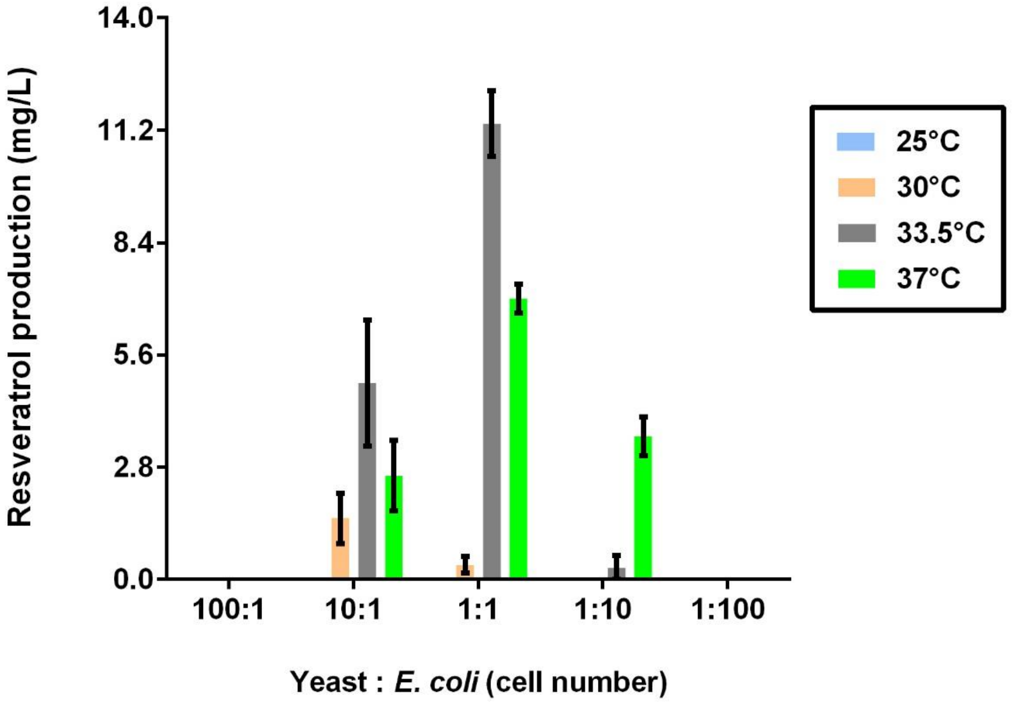
Resveratrol production (mg/L)
14.0
11.2
8.4
5.6
2.8
0.0
100:1
10:1
1:1
1:10
1:100
Yeast : *E. coli* (cell number)
25°C
30°C
33.5°C
37°C

b

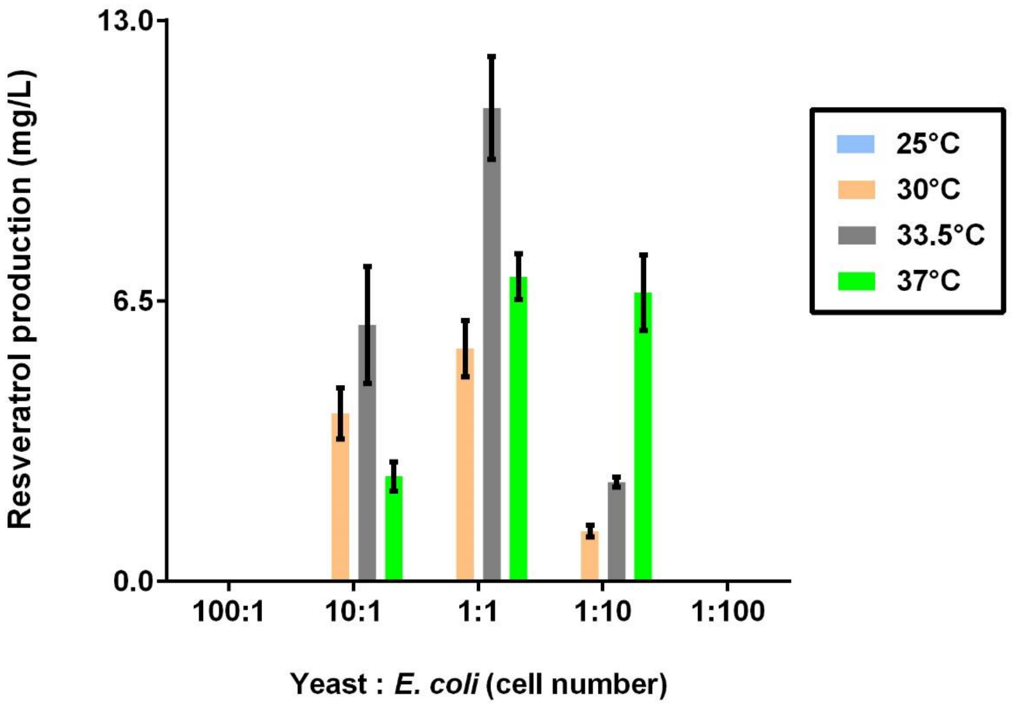
Resveratrol production (mg/L)
13.0
6.5
0.0
100:1
10:1
1:1
1:10
1:100
Yeast : *E. coli* (cell number)
25°C
30°C
33.5°C
37°C

c

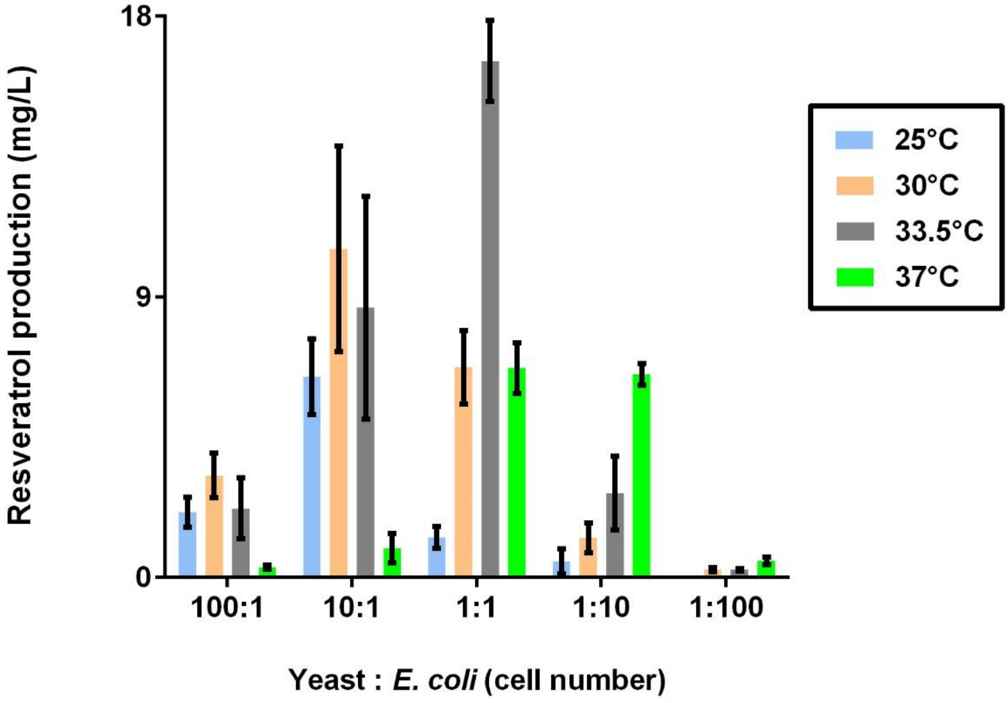

Resveratrol production (mg/L)
18
9
0
25°C
30°C
33.5°C
37°C
100:1
10:1
1:1
1:10
1:100
Yeast : *E. coli* (cell number)

d

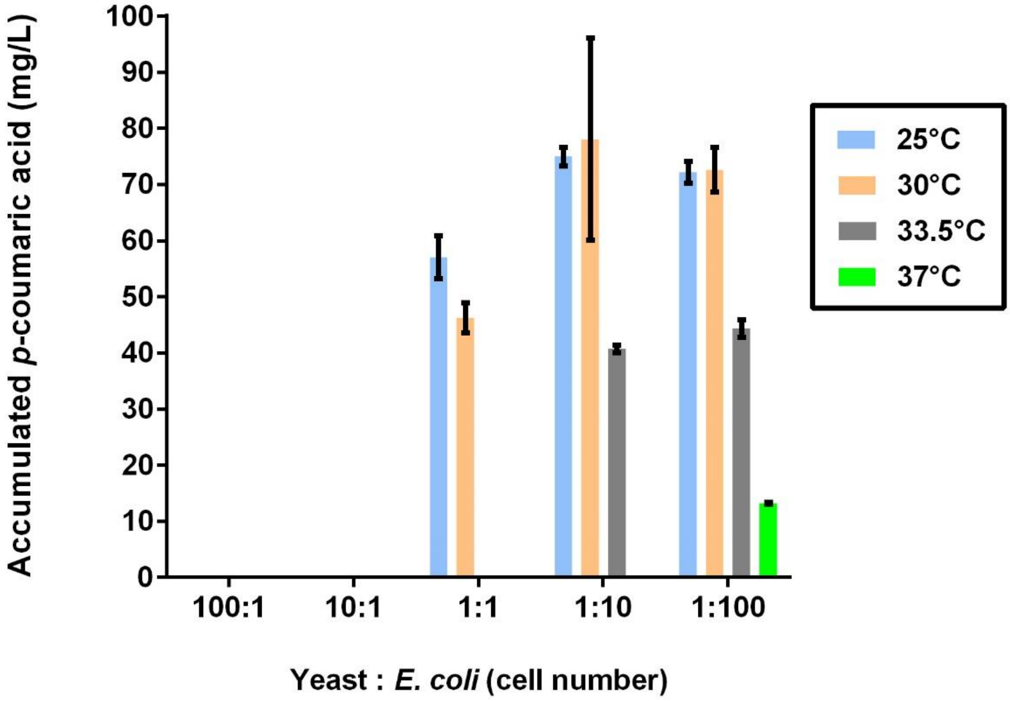

Accumulated *p*-coumaric acid (mg/L)
100
90
80
70
60
50
40
30
20
10
0
25°C
30°C
33.5°C
37°C
100:1
10:1
1:1
1:10
1:100
Yeast : *E. coli* (cell number)

e

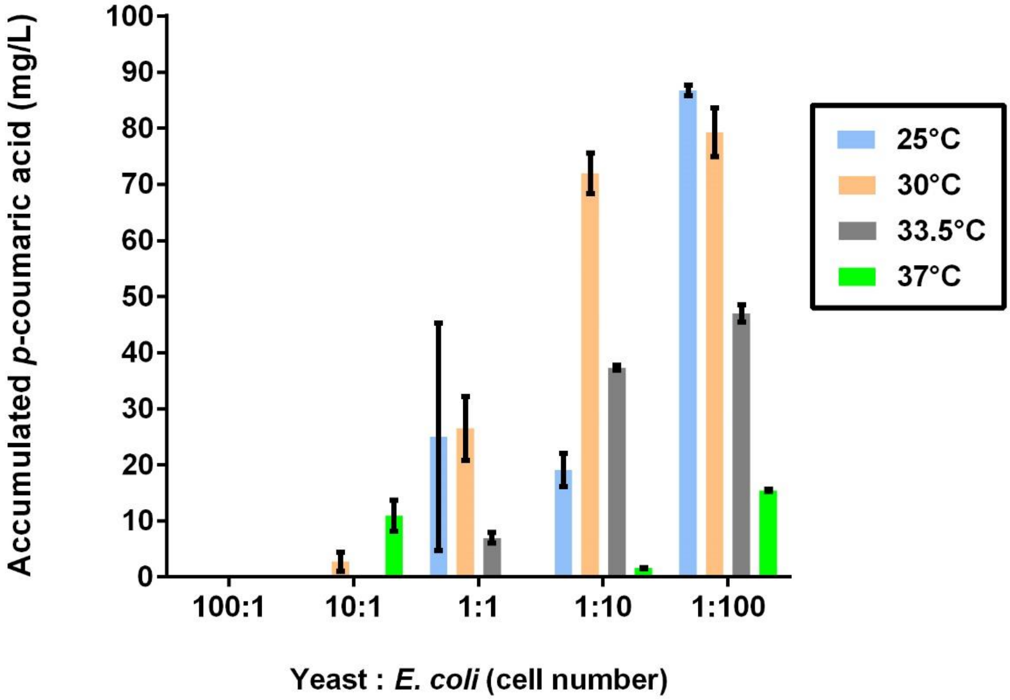
Accumulated p-coumaric acid (mg/L)
100
90
80
70
60
50
40
30
20
10
0
100:1
10:1
1:1
1:10
1:100
Yeast : E. coli (cell number)
25°C
30°C
33.5°C
37°C

f

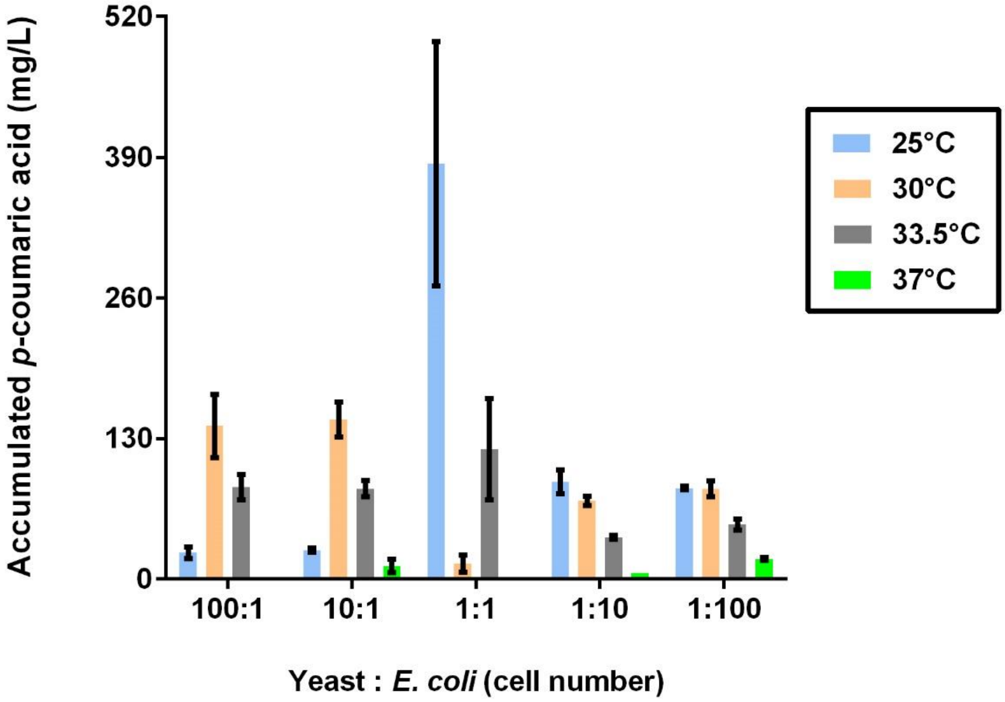
Accumulated p-coumaric acid (mg/L)
520
390
260
130
0
100:1
10:1
1:1
1:10
1:100
Yeast : E. coli (cell number)
25°C
30°C
33.5°C
37°C

Fig. S3. Investigation of the inoculation ratios of the *E. coli*-yeast co-culture and fermentation temperatures for resveratrol production. The comparison of **a-c** resveratrol production, and **d-f** accumulated *p*-coumaric acid at a test tube scale. Samples from fermentations at 20-h (**a and d**), 48-h (**b and e**) and 72-h (**c and f)** timepoints were collected for HPLC analysis. Each data point and error bar represent means and standard deviations from biological triplicates, respectively.

Table S1. List of primers used in this study.

Primer ID	Primer description	Sequence (5'->3')
P67	lppB30	CCCATCAAAAAAATATTCTCAACATAAAAAACTTTGTGTAATACTTGTAACGCTTCTAGAGATTAAAGAGGAGAAATACTAG
P68	lppB32	CCCATCAAAAAAATATTCTCAACATAAAAAACTTTGTGTAATACTTGTAACGCTTCTAGAGTCACACAGGAAAGTACTAG
P80	lppTcXALB30f	TCTAGAGATTAAAGAGGAGAAATACTAG-ATGTTTATTGAAACCAACGTG
P81	lppTcXALB30r	CAGGCGCGCCGAGCTCGAATTCGGATCC-TTAAAACATTTTACCCACTGC
P82	pCDFlppTcXALf	GGATCCGAATTCGAGCTCG
P83	pCDFlppTcXALr	AGTTTTTTATGTTGAGAATATTTTTTTGATGGG-TGAGCGCAACGCAATTAATGTAAG
P84	lppTcXALB32f	CTTCTAGAGTCACACAGGAAAGTACTAG-ATGTTTATTGAAACCAACGTG
P85	XY032	TTGAAGCCACGCAAGTAA
P86	XY033	TTCGACCTAGCCAGAATG
P87	XY034	CTGCGGTGAATTTGACCA
P88	XY035	GAGACTTGTGTTGATGCC

P89	XY036	CAACTACGAGAGCGATCG
P90	XY037	ACGTAAAGTCAGGCAAGG
P91	XY038	TCCTTCTCAGATGAGGACA
P92	XY039	CTGTGAGCCTCTTACCTG

The underlined sequence indicates that the nucleotides used to be annealed to the template for PCR amplification.

Table S2. Sequences of codon-optimized genes used in this study.

Description	Sequence (5'->3')
T. cutaneum TAL (codon-optimized for *E. coli*)	atgtttattgaaaccaacgtggcaaaaccggctagcacgaaagcgatgaatgccggctctgcaaaagcggccccggtcgaaccgttcgctacctatgcgcatagtcaggccaccaaaacggt<br>gtccatcgatggccacacgatgaaagttggtgacgtggttgctgttgcgcgtcatggcgcgaaagttgaactggcagctagtgttgctggtccggtccgtgcgtccgtggattttaaagaaagca<br>aaaaacacacctcgatttatggcgtgaccacgggtttcggcggttcagccgatacccgtacgtcggacacggaagcactgcagatctctctgctggaacatcaactgtgcggctttctgccgac<br>cgatgcgacgtacgagggtatgctgctggcggccatgccgattccgatcgtgcgtggtgcgatggcggtccgtgtgaacagctgtgttcgtggccactctggtgttcgcctggaagtcctgca<br>gagctttgccgatttcattaatcgtggtctggttccgtgcgtcccgctgcgtggtaccatcagtgcatccggtgacctgtcaccgctgtcgtatattgctggcgcgatctgtggtcatccggatgtta<br>aagtcttcgacaccgcagcttcaccgccgaccgttctgacgtcgccggaagcaattgcaaaatatggcctgaaaaccgtcaaactggcgagcaaagaaggcctgggtctggttaacggtacg<br>gcagtctctgcggcggcaggtgctctggcactgtacgatgccgaatgcctggcaatcatgagtcagaccaatacggtgctgaccgttgaagctctggacggccatgttggttcctttgcaccgtt<br>cattcaggaaatccgtccgcacgcgggccaaattgaagctgcgcgtaacatccgccatatgctgggcggttcaaaactggccgtgcacgaagaatcggaactgctggctgatcaggacgcg<br>ggtattctgcgtcaagatcgctacgccctgcgtaccagtgcacagtggatcggtccgcaactggaagccctgggtctggcacgccagcaaattgaaacggaactgaactccaccacggataa<br>tccgctgatcgacgtggaaggcggtatgtttcatcacggcggtaacttccaggcgatggcggtcaccagtgctatggattccgcgcgcattgtgctgcagaatctgggtaaactgtcatttgcac<br>aagtgaccgaactgatcaactgcgaaatgaatcatggcctgccgtcgaacctggcgggtagcgaaccgtctaccaattatcattgtaaaggcctggatattcactgcggtgcctactgtgcaga<br>actgggctttctggcgaacccgatgagcaatcatgttcagtctaccgaaatgcacaaccagagcgtgaacagcatggcgttcgcaagcgcacgtcgcacgatggaagcgaacgaagttctga<br>gtctgctgctgggttcccagatgtattgtgctacccaagcgctggatctgcgcgtcatggaagtgaaatttaaaatggccattgtgaaactgctgaatgaaaccctgacgaaacattttgccgcatt<br>cctgaccccggaacagctggcgaaactgaacacccacgctgcgatcacgctgtacaaacgtctgaatcagaccccgtcatgggattcggcaccgcgctttgaagacgccgcaaaacatctg

	gtgggcgttattatggatgcgctgatggttaacgatgacatcaccgacctgacgaatctgccgaaatggaagaaagaatttgccaaagaagcaggtaacctgtatcgtagcattctggtggctac<br>cacggcggatggccgcaatgacctggaaccggccgaatatctgggtcagacccgtgccgtgtacgaagcagttcgcagcgaactgggcgtcaaagtgcgtcgcggtgatgttgcggaagg<br>caaaagcggtaaatctattggcagctctgtcgctaaaatcgtggaagcaatgcgtgacggtcgcctgatgggtgcagtgggtaaaatgttttaa
A. thaliana 4CL<br>(codon-optimized<br>for *S. cerevisiae*)	atggctccacaagaacaagctgtttcccaagttatggaaaagcaatctaacaacaacaactccgacgtcatcttcagatctaaattgccagatatctacatcccaaaccacttgtcattgcacgatt<br>acatcttccaaaacatctctgaattcgctaccaagccatgtttgattaacggtccaactggtcatgtttacacctactctgatgttcacgttatctccagacaaattgctgctaactttcacaagttgggt<br>gtcaatcaaaacgatgtcgtcatgttgttattgccaaactgtccagaattcgtcttgtctttttggctgcttcttttagaggtgctactgctacagctgctaatccatttttactccagctgaaattgctaa<br>gcaagctaaggcttctaacaccaagttgattattaccgaagctagatacgttgacaagatcaagccattgcaaaatgatgatggtgttgttatcgtctgcatcgatgataatgaatccgttccaattcc<br>agaaggttgtttgagattcactgaattgactcaatctactaccgaagcctccgaagttattgattccgttgaaatttctccagatgatgttgttgctttgccatactcttcaggtactactggtttgccaaa<br>aggtgttatgttgactcataagggtttggttacatccgttgctcaacaagttgatggtgaaaatccaaacttgtacttccactccgatgatgtcattttgtgtgttttgccaatgttccatatctacgccttg<br>aactctattatgttgtgcggtttgagagttggtgctgctattttgattatgccaaagttcgaaatcaatttgttgttggaattgatccaaagatgcaaggttactgttgctccaatggttccaccaatagttt<br>tggctattgctaagtcctctgaaaccgaaaagtacgacttgtcctctatcagagttgttaagtcaggtgctgctccattgggtaaagaattagaagatgctgttaacgccaagttcccaaatgctaaa<br>ttgggtcaaggttacggtatgactgaagctggtccagttttagctatgtctttgggttttgctaaagaaccattcccagtaaaatctggtgcttgtggtacagttgttagaaacgctgaaatgaagatc<br>gttgatccagatactggtgactccttgtctagaaatcaaccaggtgaaatctgcatcagaggtcatcaaattatgaagggttacttgaacaatccagctgctactgcagaaaccattgataaggatg<br>gttggttgcatacaggtgatattggtttgattgatgacgacgacgaattattcatcgttgatagattgaaagaattgatcaagtacaagggtttccaagttgctccagcagaattggaagctttgttgat<br>tggtcatccagatattaccgatgttgctgttgttgcaatgaaggaagaagctgctggtgaagttccagttgcttttgttgtcaaatccaaggactctgaattgtccgaagatgatgtaaagcaattcgt<br>cagtaagcaagtcgttttctacaagagaatcaacaaggttttcttcaccgaatccattccaaaagctccatctggtaagatcttgagaaaagatttgagagccaagttggctaacggtttgtga

V. vinifera STS (codon-optimized for *S. cerevisiae*)	atggcttccgttgaagaattcagaaacgctcaaagagctaaaggtccagctactattttggctattggtactgctactccagatcattgtgtttaccaatctgattacgccgactactacttcagagtta<br>ctaagtctgaacacatgaccgaattgaagaaaaagttcaacagaatctgcgacaagtccatgatcaagaagagatatatccacttgaccgaagaaatgttggaagaacatccaaacattggtgct<br>tatatggctccatccttgaacatcagacaagaaattatcactgccgaagttccaagattgggtagagatgctgctttgaaggctttgaaagaatggggtcaacctaagtctaagatcacccatttgg<br>ttttctgtactacctctggtgttgaaatgccaggtgctgattacaaattggctaacttgttgggtttggaaacctccgttagaagagttatgttgtaccatcaaggttgttatgctggtggtactgttttga<br>gaactgctaaagatttggctgaaaacaatgctggtgctagagttttggttgtttgctctgaaattaccgttgttactttcagaggtccatctgaagatgctttggattctttggttggtcaagctttgtttgg<br>tgatggttcttctgctgttatagttggttctgatccagatgtctctatcgaaagacctttgttccaattggtttctgctgctcaaactttcattccaaattctgctggtgcaattgctggtaacttgagagaa<br>gttggtttgacttttcatttgtggccaaacgttccaactttgatctccgaaaacattgaaaagtgtttgacccaagctttcgatccattgggtatttctgattggaattccttgttctggattgctcatccag<br>gtggtccagcaattttggatgctgttgaagctaaattgaacttggaaaagaagaagttggaagccaccagacatgttttgtctgaatacggtaatatgtcctctgcttgcgttttgttcattttggacga<br>aatgagaaaaaagtccttgaagggtgaaaaggctactactggtgaaggtttggattggggtgttttgttcggttttggtccaggtttgactattgaaactgttgtcttgcattctgttccaaccgttacc<br>aattga
